# DDX17 promotes hepatocellular carcinoma progression via inhibiting Klf4 transcriptional activity

**DOI:** 10.1038/s41419-019-2044-9

**Published:** 2019-10-25

**Authors:** Ying Xue, Xuebing Jia, Changcan Li, Ke Zhang, Lei Li, Jinhuan Wu, Jian Yuan, Qi Li

**Affiliations:** 1 Department of oncology, Shanghai General Hospital, Shanghai Jiao Tong University School of Medicine, 100 Haining Road, Hongkou, 200080 Shanghai, China; 2 Department of General Surgery, Tongji Hospital, Tongji University School of Medicine, 200065 Shanghai, China; 3 Clinical Medicine, Xinxiang Medical School, 453003 Henan, China; 4 Research Center for Translational Medicine, East Hospital, Tongji University School of Medicine, 200120 Shanghai, China; 5 Tianjin Medical University, School of Biomedical Engineering and Technology, 300070 Tianjin, China; 6 Key Laboratory of Arrhythmias of the Ministry of Education of China, East Hospital, Tongji University School of Medicine, 200120 Shanghai, China; 7 Department of Oncology, Mayo Clinic, Rochester, MN 55905 USA; 8 Department of Molecular Pharmacology and Experimental Therapeutics, Mayo Clinic, Rochester, MN 55905 USA

**Keywords:** Oncogenes, Tumour biomarkers

## Abstract

DEAD box RNA helicase 17 (DDX17) is a transcriptional regulator of several transcription factors, which is more appreciated than its role in RNA metabolism. However, prognostic value and biofunction of DDX17 in HCC remain unclear. Illuminating the mechanism underlying the regulating HCC progression by DDX17 may contribute to therapeutic strategies. In our study, we report for the first time that DDX17 was overexpressed in HCC specimens by using The Cancer Genome Atlas (TCGA) and immunohistochemistry (IHC) and correlated to clinical pathological characteristics and patients’ survival. In vitro, DDX17 was ascertained to alter HCC migratory and invasive capacities after overexpression and knockdown in HCC cell lines. Moreover, by performing co-immunoprecipitation (Co-IP) and GST-pull down assay, the physical association between DDX17 and Klf4 was discovered and validated. Additionally, DDX17 could modulate expressions of Klf4 target genes including E-cadherin, MMP2 by inhibiting the promoter activity. The potent correlation between DDX17 and Klf4 target gene expressions was further appraised by a same set of 30 HCC tissues. Besides, we discovered that DDX17 could not deploy its function in regulating Klf4 target gene expressions and HCC progression in Klf4-depletion condition. Intriguingly, DDX17 failed to interact with Klf4 once the zinc-finger domain was deleted and inhibited the binding of Klf4 on MMP-2 promoter. Collectively, our study enucleates novel mechanism of DDX17-mediated oncogenesis by suppressing the transcriptional activity of Klf4 thus is likely to be a therapeutic target in HCC.

## Introduction

Hepatocellular carcinoma (HCC) ranks as the fifth and seventh most common malignancy globally in men and women, respectively, also is the second leading cause of cancer-related deaths^1^. Besides, almost 80 hundred thousand new cases of HCC occurred in 2018 worldwide, surprisingly among them nearly 79 hundred thousand deaths was caused by HCC^1^, which definitely denotes that the incidence, as well as mortality of HCC have not been decreased although substantial improvement through treatment strategies is achieved. Therefore, pursuing for novel biomarkers with high sensitivity and specificity is so challenging that it requires urgent research.

DDX17 (DEAD Asp-Glu-Ala-Asp) box helicase 17, also known as p72, is a typical member of DEAD box family and it gained attention for its role as a kind of RNA helicases^2^. Besides, it has become increasingly clear that DDX17 acts as a transcription factors (TFs)-associated protein, such as NFAT-5, HDAC1, SOX2, and beta-catenin^3–6^, and exhibits its function in various tumors. Aberrant DDX17 expression was found in colon^7^ and lung cancers, which promotes tumor progression^3,7^. However, the landscape of DDX17 in HCC involving its functional role and molecular mechanisms in the context of oncogenesis has never been deciphered.

Krüppel-like factor 4 (Klf4, GKLF) is a well-known TF pivotal for the maintenance of the pluripotency stem cell state in embryonic stem cells (ESC)^8^. Loss of Klf4 expression is well established in different cancers^9,10^. Our previous study confirms that Klf4 is also deregulated in HCC and could regulate numerous cancer cell processes^11,12^. Corroborated evidence suggests the presence of Cys_2_/His_2_zinc-finger motifs in carboxy-terminal domain of Klf4 confers preferential binding to GC/GT-rich or CACCC element sequences in its target gene promoter and enhancer regions followed by expression changes of broad array of genes covering cell cycle and proliferation, such as p21, p27, p57^13–15^, and tumor metastasis like matrix metalloproteinase 2 (MMP-2) and E-cadherin^16,17^. In our study, we evaluated the prognostic value of DDX17 in HCC and explored its effect on HCC migration and invasion. Besides, we went deeper insight into the molecular mechanism of DDX17 in HCC progression, which unveiled the interaction between DDX17 and Klf4 in regulating HCC metastasis.

## Results

### DDX17 is a potential biomarker for HCC according to TCGA

**Fig. 1:**
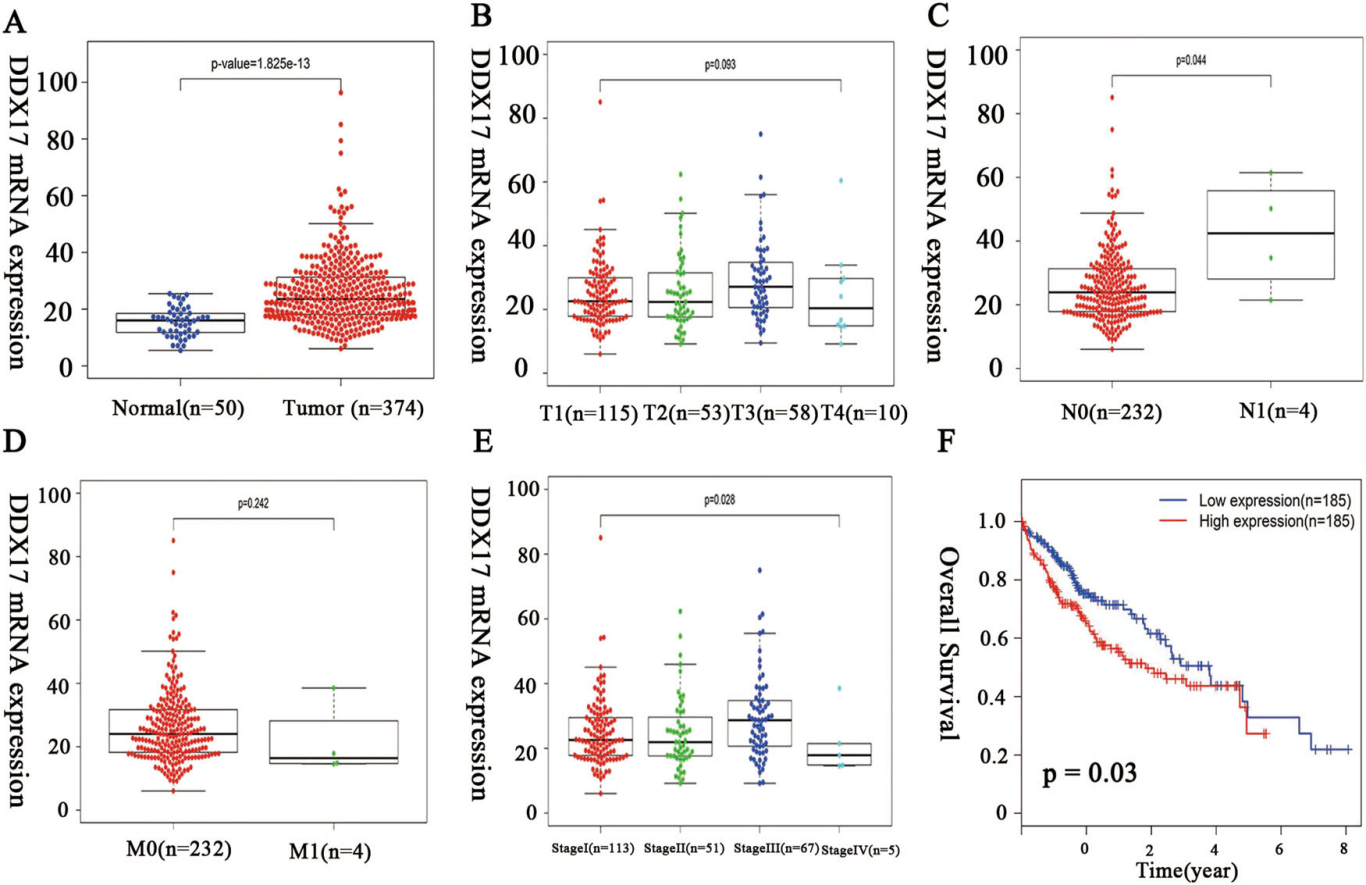
DDX17 is a potential biomarker for hepatocellular carcinoma (HCC) evaluated by The Cancer Genome Atlas (TCGA). **a** Expression level of DDX17 in hepatocellular carcinoma was elevated in HCC tumor tissues compared to normal tissue. **b** Expression of DDX17 presented more upregulated in T2, T3 stage compared to that in T1. **c** Expression of DDX17 in HCC patients with N1 stage was remarkedly increased compared that in N0 stage. **d** There was no significant difference of DDX17 expression between M1 stages and M0 stage in HCC patients. **e** Expression of DDX17 was elevated gradually as HCC progressed to stage II and stage III (The tumor stage was according to AJCC. T: Tumor, representing the extent of the primary tumor; N: lymph node, representing the presence or absence of regional lymph node metastasis; M: Metastasis, representing the presence or absence of distant metastases). **f** OS curve of HCC patients based on DDX17 expression (*p* = 0.03)

We first utilized TCGA database to access DDX17 mRNA expression and its association with HCC prognosis. As shown in Fig. 1a, DDX17 was remarkedly overexpressed in HCC compared to normal tissue at transcriptional level (*p* < 0.001). Then, we further evaluated its association with clinical pathological characteristics. As shown in Fig. 1b–e, although there was no significance between DDX17 expression and tumor size and M stage, DDX17 was correlated to tumor stage and lymphatic metastasis. Since the samples of M1 stage, N1 stage, and stage IV from TCGA were small, so that it was not sufficient to demonstrate correlation between DDX17 and stage progression, N metastasis. But potent correlation between DDX17 expression and overall survival of HCC patients was found (Fig. 1f, *p* = 0.03). Therefore, we assumed that non-significance between DDX17 and tumor size or distant metastasis was probably due to lack of large samples, which requires further research to validate.

### DDX17 is upregulated in HCC specimens and predicts poor clinical outcomes in HCC patients

**Fig. 2:**
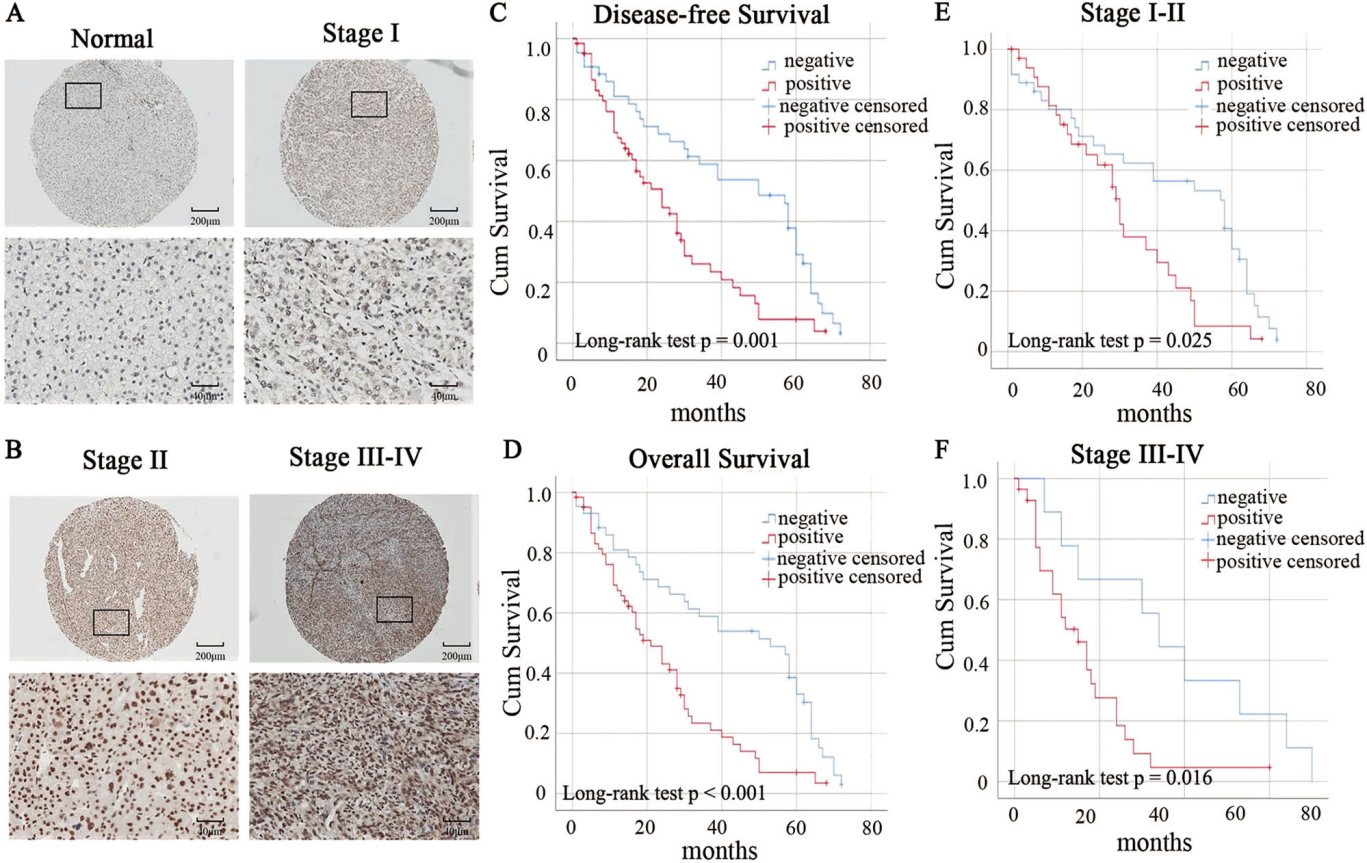
DDX17 is upregulated in HCC tissues and was associated with poor prognosis in HCC patients. **a**, **b** Expression of DDX17 increased as HCC progressed to more advanced stages. DDX17 protein expression was accessed by IHC analysis in 105 paired HCC specimens. The IHC score of DDX17 was calculated as the staining intensity (0, 1, 2, or 3) × the staining extent (0–100%). **c** DFS curve of HCC patients based on DDX17 expression according to Kaplan–Meier analysis. **d** OS curve of HCC patients based on DDX17 expression according to Kaplan–Meier analysis. Patients with high levels of DDX17 were prominently associated with poor DFS (*p* = 0.001) and OS (*p* *<* 0.001). **e**, **f** OS curve of HCC patients with different DDX17 expression was further analyzed according to tumor stage. *p *<* 0.05

In order to validate that correlation between DDX17 expression and HCC progression, IHC was performed to evaluate the DDX17 protein expression in 105 human HCC specimens and substantially overexpressed DDX17 protein was found in HCC specimens compared to adjacent normal tissue (Table 1, *p* < 0.001) and results also showed that the expression of DDX17 was increased significantly as HCC progressed to more advanced stage (Fig. 2a, b). Moreover, the association between DDX17 expression and clinical pathological characteristics are shown in Table 2. The over-expression of DDX17 was strikingly correlated to tumor size (*p* = 0.03), nodal involvement (N stage) (*p* = 0.019), distant metastasis (M stage) (*p* = 0.037), tumor differentiation (*p* = 0.0012), and American Joint Committee on Cancer (AJCC) stage (*p* < 0.001). However, there were no associations between DDX17 expression and patient age and gender.

**Table 1:** Expression of DDX17 protein in paired normal tissues and HCC tissues

Tissue sample	*n*	DDX17 expression		*p*-value
		Negative	Positive	
Normal tissue	105	62	43	<0.001^a^
Tumor tissue	105	31	74	

^a^Implies statistical difference, *p* value is based on the chi-square test

**Table 2:** Correlation between DDX17 expression and clinicopathological characteristics

	Total (*n* = 105)	DDX17 protein expression		*p-*value
		Negative (*n*)	Positive (*n*)	
Age, years				
≤52	59	15	44	0.318
>52	46	17	29	
Gender				
Male	75	34	41	0.949
Female	30	10	20	
Tumor size				
>5 cm	61	21	40	0.030*
≤5 cm	44	24	19	
N stage				
N0	90	41	49	
N1	15	2	13	0.019*
M stage				
M0	97	32	65	
M1	8	1	7	0.037*
AJCC stage				
I + II	65	35	30	<0.001*
III + IV	40	8	32	
Differentiation				
Well	8	7	1	0.002*
Moderate	60	27	33	
Poor	37	9	28	

*p < 0.05 indicates a significant association among the variables

Besides, Kaplan–Meier curves with a log-rank test for DFS and OS were performed to elucidate the relationship between DDX17 expression and patients’ survival in HCC in TMA with 105 patients. Our results revealed that high expression of DDX17 was associated with shorter DFS compared with lower DDX17 expression (Fig. 2c, *p* = 0.001). Besides, high expression level of DDX17 was associated with a trend towards poor OS (Fig. 2d, *p* < 0.001). In addition, further OS analysis was performed according to tumor stage, and results manifested that patients who were in stage I–II or stage III–IV, with higher DDX17 expression had worse outcome than those with lower DDX17 expression (Fig. 2e, f). The obtained results revealed DDX17 was a potential prognostic marker for HCC.

### DDX17 promotes HCC migration and invasion in vitro

**Fig. 3:**
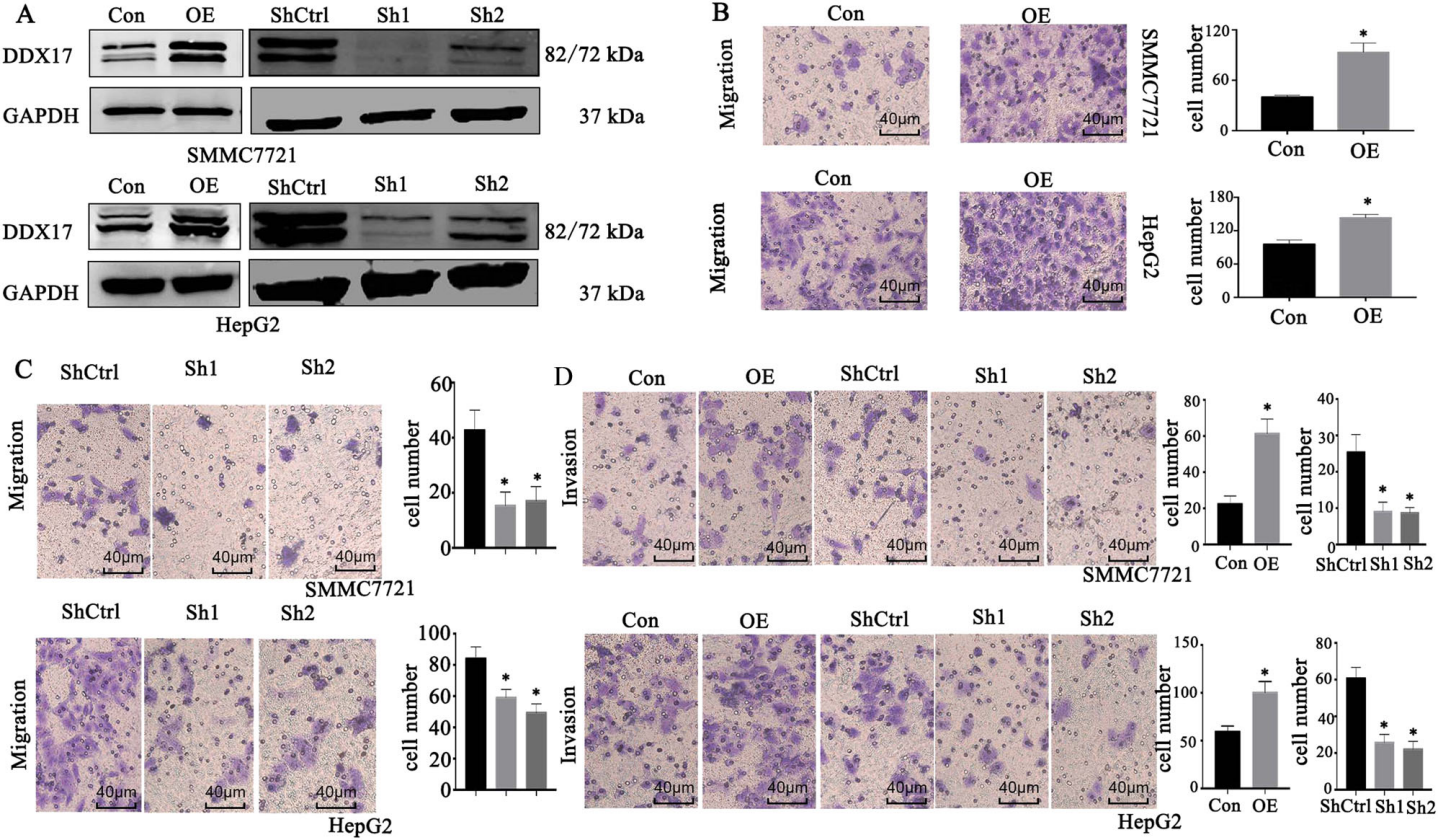
DDX17 promotes HCC migration and invasion in vitro. **a**, **b** Western blotting was used to access DDX17 expression after transfected with DDX17 plasmid or DDX17-shRNA in SMMC7721 and HepG2. **c**, **d** The migratory ability in indicated cells was detected by Transwell assay after DDX17 overexpression and knockdown separately. **e** The invasive ability in indicated cells was detected by Transwell assay after DDX17 overexpression and knockdown separately. *p *<* 0.05

To explore the effect on HCC migration and invasion, we constructed lentivirus-mediated DDX17 shRNA stable cells including SMMC7721 and HepG2 cells, and DDX17 plasmid was transfected transiently into both cells, which was confirmed by Western blotting (Fig. 3a). Then the migration and invasion assays were performed to investigate whether suppression or upregulation of DDX17 was capable of altering HCC cells’ migratory and invasive abilities. As shown in Fig. 3b–d, in DDX17 overexpressed-condition both SMMC7721 and HepG2 cells presented potentiating migratory and invasive capacities remarkedly, which however were blunt strongly after knockdown DDX17 in HCC cell lines.

### DDX17 interacts with Klf4 and alters Klf4 target gene expression

**Fig. 4:**
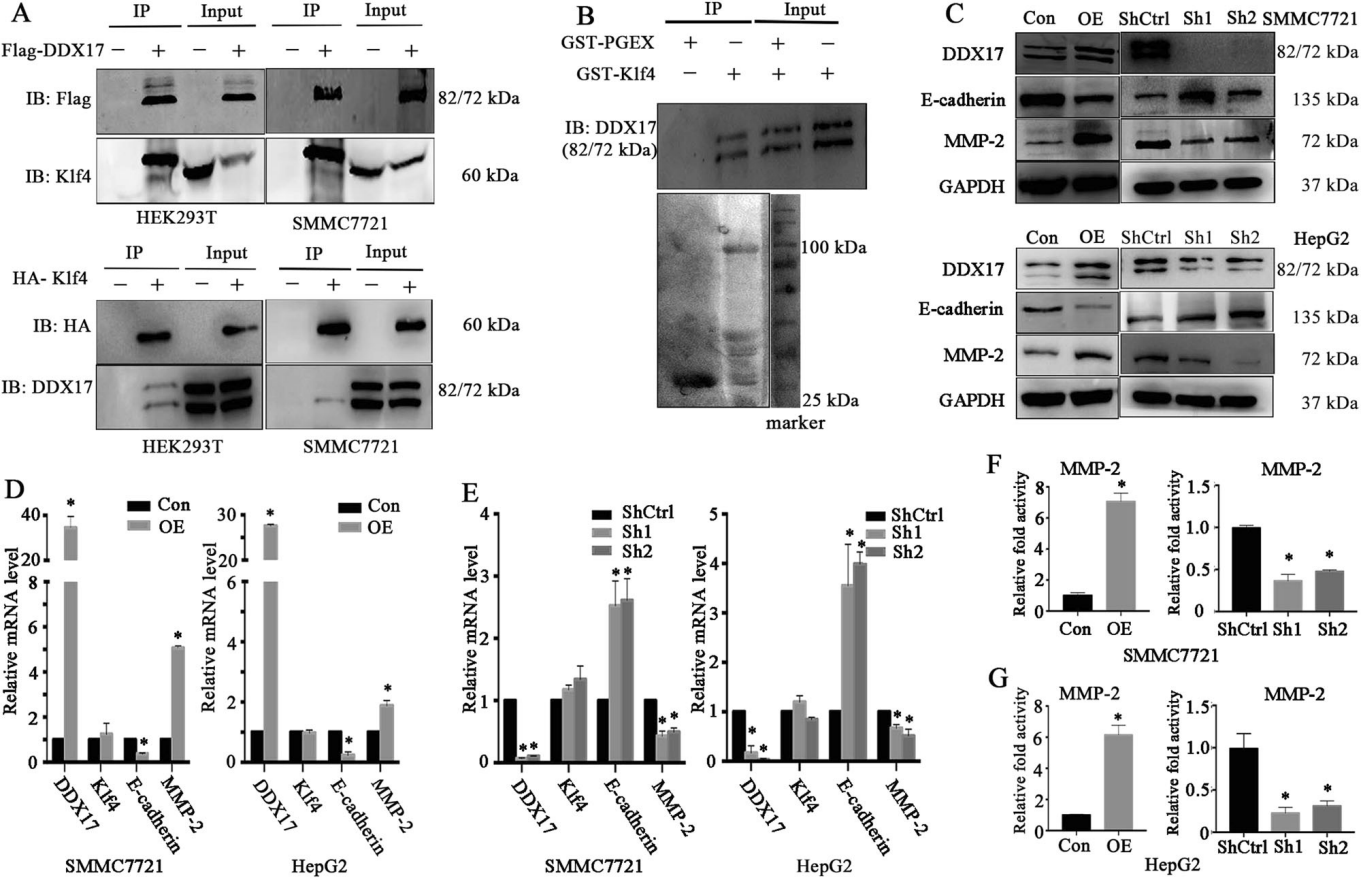
DDX17 interacts with Klf4 and alters Klf4 target gene expression. **a** Co-immunoprecipitation (IP) analysis of DDX17 and Klf4 in HEK293T and SMMC7721 cells. DDX17 and Klf4 could be reciprocally co-immunoprecipitated. **b** GST-Klf4 fusion protein was incubated with GST-beads and lysates of DDX17-transfected HEK293T cells. Western blotting was performed to probe the interacting DDX17. **c** Western blotting was performed to detect protein expression of Klf4 target genes including E-cadherin and MMP-2 in DDX17 overexpressed and depleted conditions. **d**, **e** Quantitative real-time PCR (qRT-PCR) was used to validate mRNA alterations of E-cadherin and MMP-2 in indicated cells. **f**, **g** MMP-2 promoter activity was detected by luciferase report activity in indicated cells. *p < 0.05

Our previous study found loss of Klf4 accelerated HCC progression by activating EMT process via TGF-beta-signaling pathway as well as regulation of Vitamin D^11,18^. Besides, previous studies have shown that the transcriptional activity of Klf4 could be regulated by its binding partners^19^. As DDX17 could act as co-activator or co-inhibitor of TFs, therefore, we wondered that whether DDX17 could be a regulator of Klf4 transcriptional activities. Therefore, we utilized CO-IP in HEK293T cells and SMMC7721 cells. Results revealed DDX17 and Klf4 were physically associated (Fig. 4a). To further validate their binding, GST-pull down assay was performed using purified GST-PGEX or GST-Klf4, and results showed that GST-Klf4 specifically interacted with DDX17 (Fig. 4b). Altogether, these results strongly supported the interaction of DDX17 and Klf4. To further investigate whether DDX17 affected the expression of Klf4 target genes responsible for tumor metastasis, we checked the expression of E-cadherin and MMP2, whose promoter or enhancer element is regulated by Klf4^17,20^. As shown in Fig. 4c, E-cadherin displayed lessened protein expression, while the protein expression of MMP-2 was upregulated after DDX17 upregulation. On the contrary, in DDX17-delpeted condition, the protein expression of E-cadherin was significantly elevated, while the protein expression of MMP-2 was remarkedly dropped (Fig. 4c). Furthermore, in order to demonstrate whether alteration of Klf4 target genes was ascribed to changes of transcriptional activities, the mRNA expression levels of E-cadherin and MMP-2 were quantified by qRT-PCR. As shown in Fig. 4d, e, the mRNA expression of E-cadherin and MMP-2 presented the same trend as the protein expressions after overexpression and suppression of DDX17 in HCC cell lines. To further explore whether DDX17 directly regulated the promoter activity of Klf4 target genes leading to transcriptional and translational level changes of Klf4-dependent genes, we cloned -521 to -136 base pair region of the MMP-2 promoter, which was rich in GT elements requiring Klf4 to bind on the promoter. Results from luciferase report assay revealed that the MMP-2 promoter activity was remarkedly elevated after DDX17 was overexpressed (Fig. 4f). On the contrary, the MMP-2 promoter activity was prominently decreased after knockdown of DDX17 both in HepG2 and SMMC7721 cell lines (Fig. 4g). Consequently, DDX17 modulated the expression of Klf4-dependent target genes via directly regulating its promoter activity.

### DDX17 regulates the expression of E-cadherin and MMP-2 by inducing dissociation of Klf4 on chromatin

**Fig. 5:**
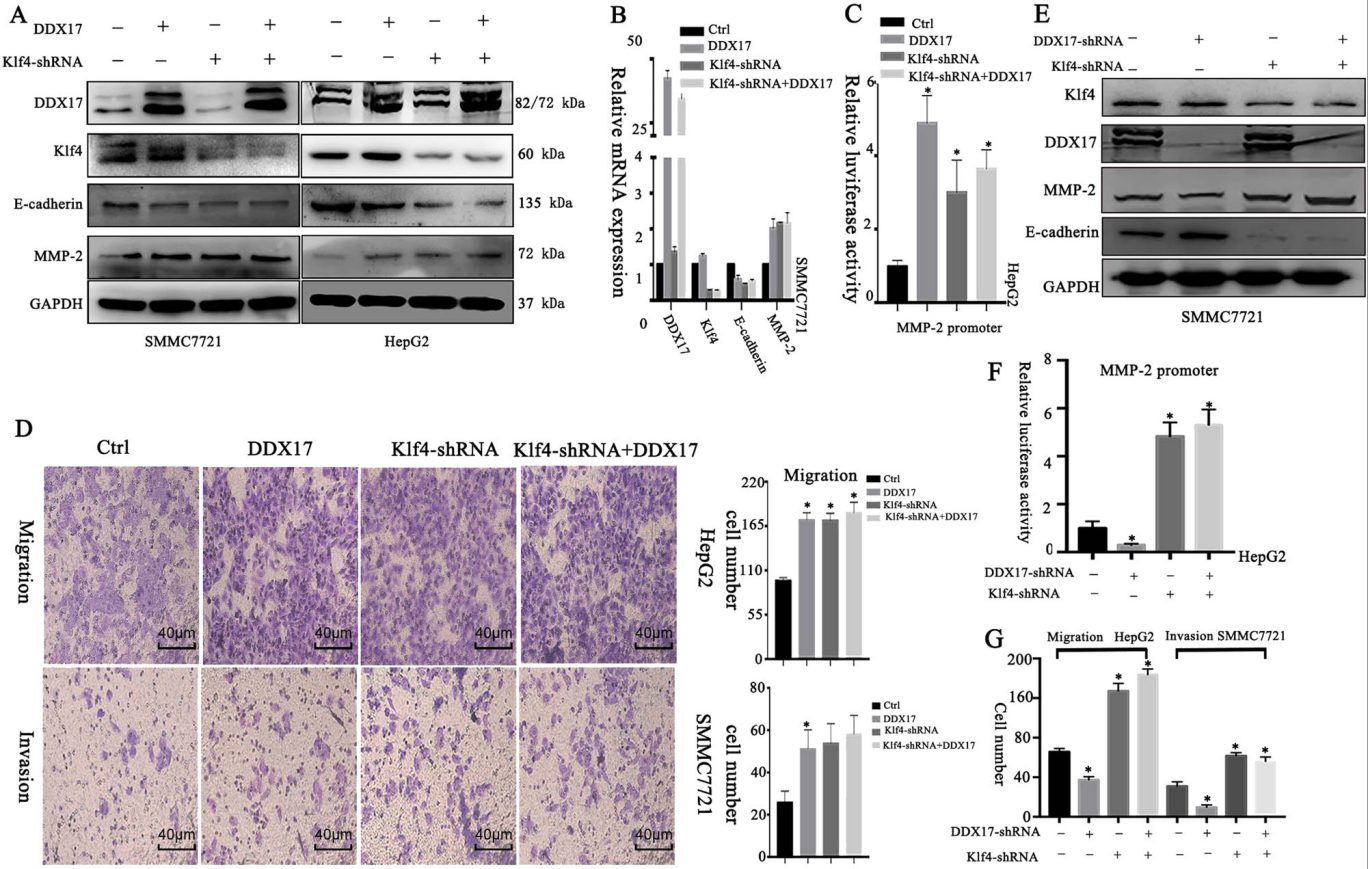
DDX17 regulates the expression of E-cadherin and MMP-2 via inhibiting Klf4 transcriptional activity. **a** Western blotting was used to analyze protein levels of E-cadherin and MMP-2 in different groups in SMMC7721 and HepG2 cells. **b** mRNA expressions of E-cadherin and MMP-2 of different groups were evaluated by qRT-PCR in SMMC772. **c** Different groups of MMP-2 promoter activity were assessed by luciferase report assay in HepG2. **d** Cell migratory and invasive capabilities were determined by Transwell assay in indicated cells. **e** Western blotting was used to analyze protein levels of E-cadherin and MMP-2 in different groups in SMMC7721 cells. **f** MMP-2 promoter activity was assessed in indicated cells. **g** Cell migratory and invasive capabilities were detected in indicated cells. *p < 0.05

To gain deeper mechanism underlying DDX17-mediated alteration of E-cadherin and MMP-2 expressions, we depleted the expression of Klf4 by shRNA in HepG2 and SMMC7721 cells, and then performed transient overexpression of DDX17. Results showed that the changes of E-cadherin and MMP-2 expressions in transcriptional and translational levels caused by Klf4 knockdown could not be further altered by DDX17 overexpression (Fig. 5a, b). As shown in Fig. 5c, MMP-2 promoter activity also presented the same trend as the protein and mRNA expressions. This highlighted that DDX17 modulated E-cadherin and MMP-2 expressions via inhibiting Klf4 transcriptional activity. Besides, DDX17 overexpression and Klf4 depletion were both able to enhance migration and invasion of HCC cells, however, DDX17 overexpression was incapable of further enhancing these abilities once Klf4 was knockdown (Fig. 5d). Furthermore, we constructed DDX17 shRNA and DDX17 shRNA + Klf4-shRNA stable HCC cell lines. Results showed loss of DDX17 could not modulate expression and MMP-2 promoter activity when Klf4 is also suppressed (Fig. 5e, f). Besides, knockdown of DDX17 indeed lost its inhibiting role in HCC progression once Klf4 is depleted (Fig. 5g; Supplementary Fig. 1a). Thus, our data suggested that DDX17 regulated HCC metastasis in a Klf4-dependent manner.

**Fig. 6:**
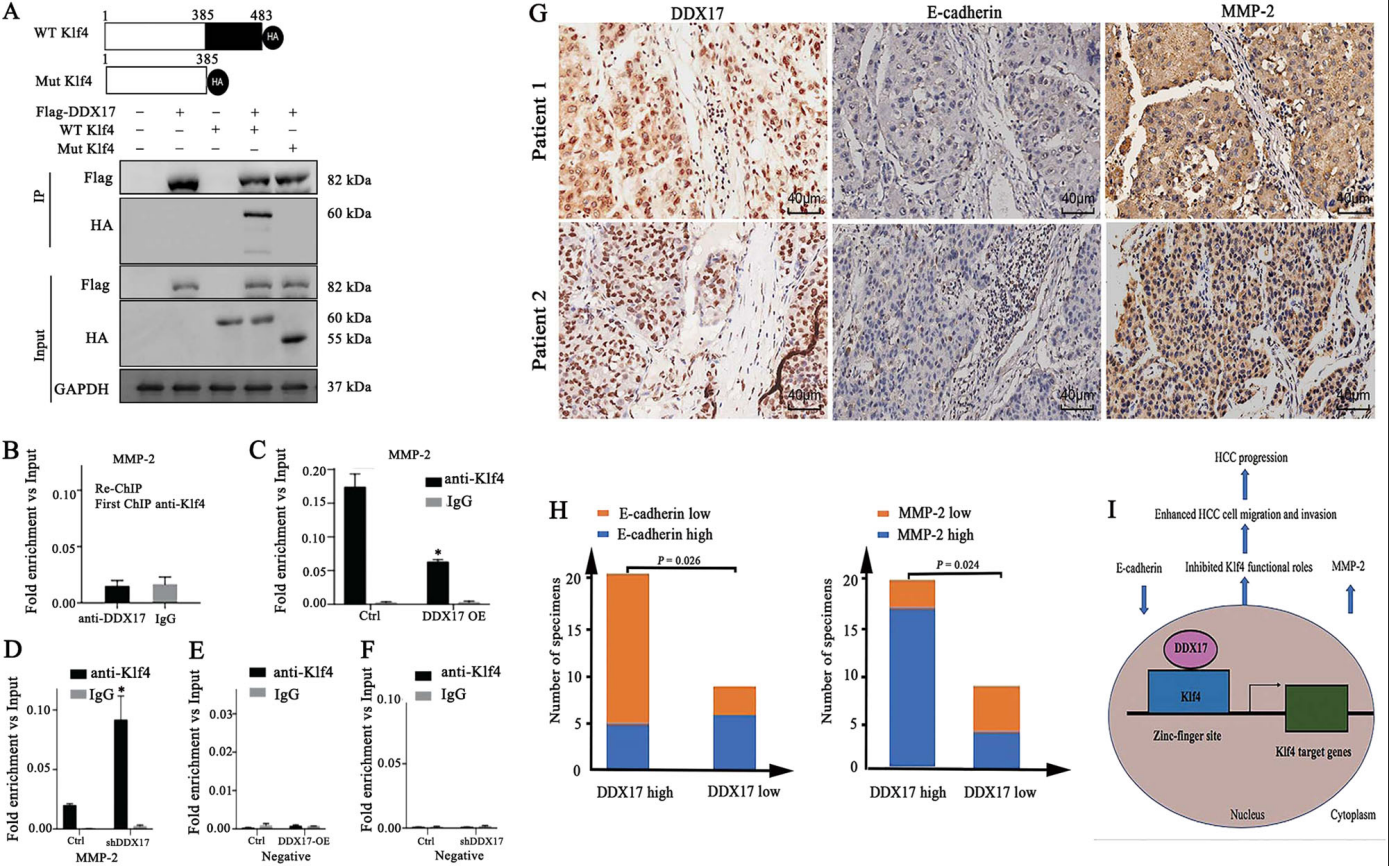
DDX17 regulates the expression of MMP-2 by inducing dissociation of Klf4 on chromatin and its expression is significantly correlated with E-cadherin and MMP-2 expressions in hepatocellular carcinoma tissues. **a** Brief description for wild type and mutant type of Klf4 and interaction between wild type/mutant type Klf4 and DDX17 was assessed using CO-IP in HEK293T cells. **b** Re-ChIP assay showed no binding between DDX17 and Klf4 at MMP-2 promoter. **c**, **d** The intensity of Klf4 binding on MMP-2 promoter was detected in DDX17 overexpression and knockdown was detected by ChIP-qPCR. **e**, **f** The intensity of Klf4 binding on negative promoter was detected by ChIP-qPCR after overexpression and knockdown of DDX17. **g** Representative images of immunohistochemical staining of DDX17, E-cadherin, and MMP-3 in tumor tissues. **h** Spearman’s correlation analysis of DDX17 and E-cadherin expression, DDX17 and MMP-2 expression. **i** Model: Diagrammatical representation, elucidating DDX17-mediated suppression of Klf4 transcriptional activity in hepatocellular carcinoma. DDX17 binds to zinc-finger domain of Klf4, subsequently inhibits Klf4 target genes including E-cadherin and MMP-2. Decreased E-cadherin expression and increased MMP-2 expression result in potentiating HCC cell migration and invasion, thus conductive to HCC progression. *p < 0.05

To explore further the relationship between DDX17 and Klf4, we generated Klf4 mutation, whose three zinc-fingers were mutant (Fig. 6a). Besides, CO-IP was performed in HEK293T cells, and results showed that DDX17 could hardly interact with mutant Klf4, which manifested that DDX17 occupied the zinc-finger domain of Klf4 thus regulated Klf4 target gene expression. Taken together, these data powerfully implicated that DDX17 regulated Klf4-mediated target genes through suppressing zinc-finger activity of Klf4. Since DDX17 was incapable to bind to Klf4 zinc-finger activity and inhibited its transcriptional activity, we wondered whether DDX17 could bind to Klf4 at DNA. Thus, Re-ChIP was performed using MMP2 promoter. However, the Re-ChIP experiment showed that the DDX17 and Klf4 did not co-localize on promoter of MMP2 (Fig. 6b). Moreover, to determine how DDX17 regulated the targets of KLF4 on chromatin, we overexpressed DDX17 in SMMC7721 cells, and determined the binding of KLF4 on the promoter of MMP2. Surprisingly, we found that the KLF4 were highly enriched in the promoter of MMP2, while DDX17 overexpression significantly reduced the binding of KLF4 on the promoter of MMP2 and vice versa (Fig. 6c, d). As a control, we detected the binding of Klf4 on a non-regulated region that was not reduced upon DDX17 silence or overexpression (Fig. 6e, f). Together, these results suggested that the DDX17 directly interact with KLF4 on chromatin and might contribute to the dissociation of KLF4 on chromatin. However, since Klf4 was also able to regulate gene expressions relevant to cell proliferation, CCK-8 and clone formation assay were performed. Results showed that DDX17 knockdown also inhibited HCC cell proliferation (Supplementary Fig. 1b–d), and results showed that loss of DDX17 remained weak ability to inhibit HCC cell proliferation although the majority of its ability disappeared in Klf4-depletion condition. This suggested that DDX17 inhibits other Klf4-target gene expression by inhibiting Klf4 transcriptional activity, but it is more complicated. Besides, this result implied that DDX17 may regulate Klf4-target gene expression in large scale, which needs further study.

### DDX17 is potently correlated with Klf4 target genes existing in HCC human tissues

To determine the clinical correlation of DDX17 with Klf4 target genes, IHC analysis was performed to detect the expression levels of DDX17, E-cadherin, and MMP-2 in the same set of HCC tissue samples. Within our expectation, the expression of DDX17 presented strong correlation with E-cadherin (*p* = 0.026) and MMP-2 (*p* = 0.024) according to Spearman’s correlation test (Fig. 6g, h). Henceforth, these data further supported our obtained results in vitro.

## Discussion

DDX17 is overexpressed in colon cancer, lung cancer, and glioma cancer, potentiating tumor cell proliferation and progression^3,7,21^. Moreover, DDX17 exerts its powerful oncogenic effect by acting as co-activator or co-inhibitor of TF such as estrogen receptor alpha (ERα)^22^. Recently, DDX17 was demonstrated to interact with β-catenin and facilitate its nuclear accumulation, thus activating β-catenin target genes, and ultimately resulting in acquired resistance of non-small cell lung cancer (NSCLC) cells to gefitinib^3^. However, in our study, we firstly reported that DDX17 was upregulated in HCC and was strongly associated with HCC clinical pathological features as well as prognosis in HCC. Thus, DDX17 was an independent prognostic factor for HCC. We also discovered that DDX17 could promote HCC cells’ progression by contributing to the migratory and invasive capacities of HCC cells. These data revealed that DDX17 might take part in indispensable signaling, consequently promoting HCC progression.

Then, our CO-IP assay results confirmed the interaction between DDX17 and Klf4 in HEK293T cells as well as in HCC cells, and GST pull down assay further demonstrated this physical interaction. Moreover, during getting deeper insight into the effects of potent interaction between DDX17 and Klf4 in HCC cell lines, we discovered that DDX17 could modulate Klf4-dependent target genes at both transcriptional and translational levels containing MMP-2 and E-cadherin, which are eligible for tumor cells to migrate and invade and are directly regulated by Klf4^16,17^. In addition, DDX17 was capable to directly potentiate MMP-2 promoter activity. Moreover, in HCC tissues, the expression of DDX17 in HCC tissues presented strong correlation with the expression of E-cadherin and MMP-2. Besides, in HCC cell lines, intriguing results were obtained that DDX17 could not further trigger protein and mRNA expression alterations of E-cadherin and MMP-2 in Klf4-depleted condition. In accordance with that, DDX17 overexpression lost its function in promoting HCC cell lines metastasis once Klf4 was depleted. These results might reveal that the process of recruiting Klf4 to its target gene’s promoter by other regulators was prevented by DDX17.

Furthermore, multitudinous evidence has proved that DDX17 usually acts as a positive or negative regulator of TF, such as SOX2, β-catenin, whose transcriptional activity is modulated by DDX17, further regulating TF target genes^6^. Thus, we proposed that transcriptional activity of Klf4 was also inhibited by DDX17. In fact, our CO-IP assay revealed that DDX17 failed to interact with Klf4 once its functional domain—zinc-finger domain was totally deleted. Therefore, DDX17 indeed impeded the process of Klf4-regulating promoter activity of its target genes. In addition, our studies found that the KLF4 and DDX17 did not co-localize on the promoter of MMP2, but we observed that DDX17 overexpression decreased the binding of KLF4 on chromatin, suggesting that the DDX17 might contribute to the dissociation of KLF4 on chromatin and vice versa. Indeed, lines of studies observed that there are many proteins that shuttles between nucleus and cytoplasm, involved in the regulation of transcription by disrupting the bindings of TFs on chromatin, this report is the first to demonstrate that DDX17 is bound to Klf4 at chromatin.

However, our study suggested that other Klf4-target genes also may be regulated by DDX17. Suppression of DDX17 barely regulated HCC cell proliferation after Klf4 was depleted. This result indicates that DDX17 may also regulate Klf4-target genes associated with proliferation, thus MMP-2 and E-cadherin are not the genes exclusively regulated by DDX17. Our hypothesis is that DDX17 may regulate plenty of Klf4 target genes and can influence the binding of Klf4 on target gene promoter or enhancer. It was noticeable that DDX17 still remained weak ability to promote HCC proliferation. This indicates that Klf4 may be not the only TF that interacts with DDX17. Because DDX17 could act as regulator of many TFs, we assumed that DDX17 may affect transcriptional activities of Klf4 and other transcriptional factors, followed by alteration of a set of their target genes expression and increased cell proliferation. However, whether all of Klf4 target genes are regulated by DDX17 needs further study to validate.

In summary, our study report for the first time that DDX17 acts as a negative regulator of Klf4 transcriptional activity in HCC and is an independent prognostic factor of HCC, which might be a potential therapeutic target. We decipher that DDX17 interacts with Klf4 and inhibits its zinc-finger activity in HCC, further being recruited by Klf4 to MMP-2 promoter and modulating their expressions, ultimately promotes HCC metastasis, which was depicted in Fig. 6i.

## Material and methods

### Cell culture and transfection

HEK293T and HCC cells (HepG2, Hep3B, and SMMC7721) were cultured as previously described^23^. The small hairpin RNA (ShRNA) was constructed using the following primers:

DDX17 shRNA1: CCCAATCTGATGTATCAGGAT;

DDX17shRNA2: GACCACAAGTTGATCCAACTA;

Klf4 shRNA: GCTCCATTACCAAGAGCTCAT.

HA-Klf4 and Flag-DDX17 expression plasmids were obtained from Shanghai Asia-Vector Biotechnology. Mutant Klf4 plasmids were constructed by PCR from HA-Klf4. The MMP2 proximal promoter (−521/−136 bp) was amplified by PCR. Transfection and stable cell lines were performed in detail as previously described^23^.

### Co-immunoprecipitation (Co-IP) and immunoblotting

HEK293T and SMMC7721 cells maintained in normal growth medium were transfected with Flag-DDX17 or HA-Klf4 plasmid for 48 h. Transfected cells were harvested and lysed. Then, Flag-M2/HA beads (Sigma-Aldrich) were used to immunoprecipitate with Flag-DDX17 or HA-Klf4, and the precipitate was washed four times using cell lysis buffer. Finally, the proteins were analyzed by Western blotting.

### GST pull-down assay

GST fusion protein containing pGEX-Klf4 or pGEX vector was induced with 0.1 mM of IPTG (Sigma-Aldrich). GST or GST-Klf4 was purified by GSTrap™ FF (GE Healthcare). Total cell lysates were incubated with GST beads (Biosciences, Sweden) overnight at 4 °C followed by four times washing using washing buffer. Finally, proteins were visible by western blot analysis.

### Luciferase reporter assay

To construct rDNA promoter-activated luciferase reporter, −521/−136 bp of rDNA promoter (the transcription start site is represented as +1) was subcloned into PGL3. Luciferase reporter assay was performed in SMMC7721 cell and HepG2 cells. Briefly, cells were seeded in 24-well plates and then were co-transfected with indicated plasmids and the rDNA-promoter luciferase reporter plasmid for 48 h. Luciferase was detected using the Dual-Luciferase assay kit (Promega). Renilla was co-transfected to normalize transfection efficiency.

### RNA isolation and quantitative real time PCR (qRT-PCR)

Total RNA was extracted from indicated cells by Trizol (Takara) according to the manufacturer’s instruction, followed by reverse-transcribed by a PrimeScript RT reagent kit (Takara). Real-time PCR was performed in triplicate using SYBR Premix Ex TaqTM (Takara) and by using an Applied Biosystems 7500 Real-time PCR system. The following primers were used:

GAPDH, forward 5′-GGCATGGACTGTGG TCATGAG-3′

GAPDH, reverse 5′-T GCACCACCAAC TGCTTAGC-3′;

DDX17, forward 5′-GATG TTTGTCCTAAACCCGTGT-3′

DDX17, reverse 5′-CC AACGGAAATCCCTGGCA-3′;

Klf4 forward 5′-CTGGTTCCGCGTGGATCCCCAGGA-3′

Klf4 reverse 5′-TCACGATGCGGCCGCTCGAGTCGACCCGG-3′;

MMP2, forward 5′-TACAGGAT CATTGGCTACACACC-3′

MMP2, reverse 5′-GGTCAC ATCGCTCCAGACT-3′;

E-cadherin, forward 5′-CGAGAGCTACACGTTCACGG-3′

E-cadherin, reverse 5′-GGGTGTCGAGGGAAAAATA GG-3′.

### Chromatin immunoprecipitation (ChIP) and Re-ChIP assays

ChIP assay was performed on SMMC7721 cells with and without the indicated treatments using a kit according to the supplier’s protocol (ChIP-IT High Sensitivity^®^ Active Motif). Klf4 was immunoprecipitated using anti-Klf4(Abcam). Pre-immune rabbit serum was used as negative control. For chromatin re-immunoprecipitation (re-ChIP) assay, the chromatin immunoprecipitation was eluted from the first ChIP with 10 mm DTT at 37 °C for 30 min and diluted 20 times with ChIP dilution buffer (1% Triton X-100, 2 mm EDTA, 20 mm Tris–HCl, pH 8.1, and 150 mm NaCl) and immunoprecipitated with the DDX17 antibody (Proteintech). The immunoprecipitated DNA was uncross-linked, subjected to proteinase K digestion, and purified using QIAquick columns (Qiagen, Valenica, CA). qPCR was performed to analyze ChIP and re-ChIP samples by using the following primers: forward, 5′-TGGCATAATGATGTGGCTGT-3′, and reverse, 5′-TTGGCTGGAAAAGGTGTAGG-3′ that would amplify the fragment encompassing the Klf4-binding site.

### Immunohistochemistry

A total of 105 HCC and paired normal tissues arrays were presented by Dr. Yu. Three continuous TMA with 30 cases of HCC specimens were freely donated by Dr. Yu. TMA was finished and relevant proteins were scored as previously described^23^.

### Transwell assay

Cells migration and invasion were accessed using transwell filter (Corning, USA) as described previously^23^. In brief, cells were fixed using 4% methyl alcohol for 15 min followed by staining via 5% crystal violet solution for additional 15 min after culturing for 48 h. Five fields of view were randomly selected and photographed using an upright Metallurgical Microscope (Leica, Germany).

### Cell counting kit-8 (CCK-8) assay

CCK-8 assay was performed to detect cell proliferation. Different groups of cells were plated in 96-well plate with 100 μl medium per well. After culture for 6, 24, 48, 72, 96 h, 120 μl medium containing 10 μl. CCK8 (Dojindo, Japan) was added to each well. Cells were cultured for another 2 h in incubator and then the absorbance was measured at a wavelength of 450 nm.

### Colony formation assay

Cells were seeded in six-well plates, with a density of 1 × 10^3^ cells per well and then cultured for 14 days. Then, the cells were fixed using 4% methyl alcohol for 15 min and stained with 5% crystal violet solution for additional 15 min. Finally, clones were counted to evaluate cell proliferation.

### Statistical analysis

All statistical analyses were implemented with the SPSS 23.0 statistical software package. t-text, chi-square text, and Fisher’s exact test were used to analyze in experimental groups. Besides, the Kaplan–Meier test was used to analyze the survival rates. Spearman’s correlation test was applied to analyze the correlation. *p*-value < 0.05 in all cases was considered statistically significant.

## Supplementary information

DDX17 promotes HCC cell proliferation
