# Supplementary material for: DDX17 promotes hepatocellular carcinoma progression via inhibiting Klf4 transcriptional activity
Source: Cell Death Dis. 2019 Oct 25;10(11):814. doi: 10.1038/s41419-019-2044-9 (PMC6814716; doi:10.1038/s41419-019-2044-9)

**Supplementary Figure 1** **DDX17 promotes HCC proliferation and metastasis. a** Representative images of Transwell assay in indicated cells. **b** Clone formation was assessed in DDX17 knockdown and control cells. **c** Clone formation was assessed in Klf4-depleted cells. **d** Cell proliferation was assessed in DDX17 knockdown and control cells. **e** Cell proliferation was assessed in Klf4-depleted cells. **p*<0.05.


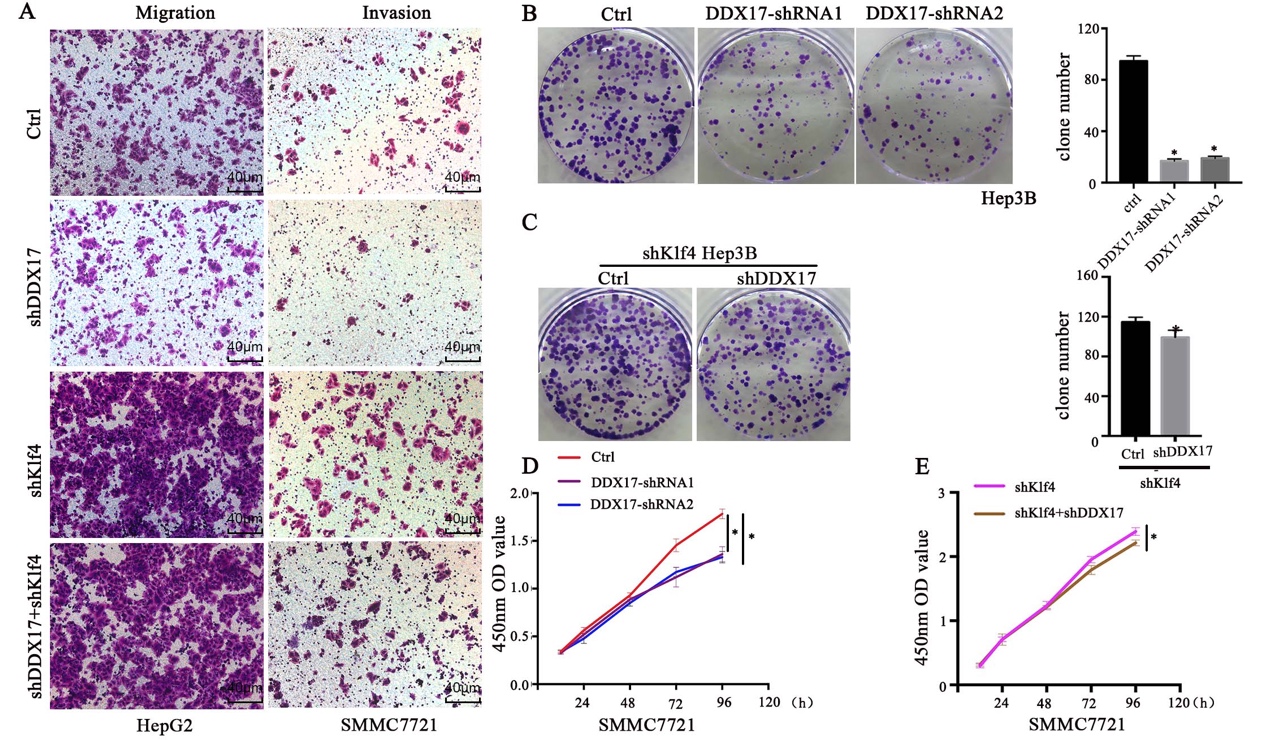

Supplement: Supplementary file 1 — DDX17 promotes HCC cell proliferation [file 41419_2019_2044_MOESM1_ESM.docx]
